# Vacuum Evaporation of High-Quality CsPbBr_3_ Thin Films for Efficient Light-Emitting Diodes

**DOI:** 10.1186/s11671-022-03708-1

**Published:** 2022-08-02

**Authors:** Tianxinyu Bai, Shenwei Wang, Liyuan Bai, Kexin Zhang, Chunyang Chu, Lixin Yi

**Affiliations:** Institute of Optoelectronic Technology, Beijing Jiaotong University, Beijing, 100044 China

**Keywords:** CsPbBr_3_, Co-evaporation, Crystallinity, Annealing, Stability

## Abstract

The all-inorganic lead halide perovskite has become a very promising optoelectronic material due to its excellent optical and electrical properties. Device performances are currently hindered by crystallinity of the films and environmental stability. Here, we adopted dual-source co-evaporation method to prepare CsPbBr_3_ films. By adjusting and controlling the co-evaporation ratio and substrate temperature, we obtained CsPbBr_3_ films with large grain sizes and uniform morphology. Films with smooth surfaces and large grains exhibit properties such as efficient photon capture, fast carrier transport, and suppressed ion migration. Therefore, in this paper, by refining the annealing conditions, the effects of annealing temperature and time on the films were studied in detail. The CsPbBr_3_ films were annealed under suitable annealing temperature and time in ambient air, and films with high quality and crystallinity and average grain size up to ~ 2.5 μm could maintain stability in ambient air for 130 days. The corresponding LEDs show the full width at half maximum (FWHM) of the green EL spectrum is as narrow as 18 nm, and the devices have a low turn-on voltage *V*_T_ ~ 3 V and can work continuously for 12 h in ambient air.

## Introduction

Metal halide perovskites of CsPbX_3_ (X = Br, I, and Cl) have become a research hotspot in recent years due to their many advantages, such as excellent thermal stability [1], extremely narrow emission bandwidth, and tunable band gap [2], and these exceptional properties have also made perovskites suitable for a variety of optoelectronic devices [3, 4], especially light-emitting diodes (LEDs) [5–9] and solar cells [10–12].

The preparation of high-quality inorganic CsPbBr_3_ perovskite films by a specific method is of great significance for improving the device performance. Researchers usually use solution method to prepare perovskites films. Because of the poor solubility of CsX in the precursor solution, it would be an obstacle for the reproducibility of smooth surface and full coverage CsPbBr_3_ films [13]; when the solvent is removed by heating, the residual solvent is prone to leak current caused by defects such as pinholes [14], which adversely affects the performance of the device. Therefore, scientists have made a lot of efforts in films optimization, mainly by adding acid or organic ammonium ligands [15–18], interfacial energy modification [19], optimizing precursor solutions [20], and other methods to improve the surface morphology of the films. Cao and co-workers introduced amino-acid additives into the perovskite precursor solutions [21], and the additives can effectively passivate perovskite surface defects and reduce non-radiative recombination, so the prepared perovskite films with the sharp and strong diffraction peaks indicate that the crystallization quality has been improved. Rogach and co-workers used cesium trifluoroacetate (CsTFA) as the cesium source, instead of the commonly used cesium bromide (CsBr) [22]. The CsTFA-based precursor produced smooth, fully covered perovskite films with improved photoluminescence quantum yield.

The vacuum evaporation method can easily deposit multilayer films with good flatness and morphology [23], however, which is less reported. Here, we report the use of dual-source vacuum co-evaporation to prepare CsPbBr_3_ films. During the lengthy deposition process (usually 2–3 h), it is difficult to maintain a correct ratio of evaporation rates of source materials throughout the process because the experimental conditions fluctuate [24], so adjusting and controlling the ratio of evaporation rates of source materials is key of co-evaporation. By adjusting the co-evaporation ratio and substrate temperature, highly phase-pure CsPbBr_3_ is successfully fabricated and the surface morphology of the films was well regulated. Moreover, perovskite films with large grain sizes have fewer internal grain boundaries. Therefore, large grain sizes, uniform density, high crystallinity are properties of high-quality perovskite films. Devices of perovskite thin films with these properties usually have higher efficiency and stability, so how to prepare the thin films with large grain sizes has become one of the hot spots of scientific researchers. Here, CsPbBr_3_ films were annealed under suitable annealing conditions in ambient air. It should be pointed out that in most of the other studies, the annealing process of vacuum-deposited inorganic CsPbBr_3_ films was carried out under nitrogen atmosphere [10, 13, 25, 26]. In addition, the CsPbBr_3_ films achieved high luminescent intensity and smooth morphology and the average grain sizes over 2.5 μm. What’s more, the films had superior stability, which maintained optical properties in ambient air for more than four months. The corresponding LEDs showed the full width at half maximum (FWHM) of the green EL spectrum was as narrow as 18 nm, and the devices had a low turn-on voltage *V*_T_ ~ 3 V and can work continuously for 12 h in ambient air, a maximum luminance (*L*_max_) of 252 cd m^−2^. It provides a good foundation for its application in later device.

## Experimental

The substrates were cleaned by deionized water, detergent, acetone, and absolute ethanol and then dried by flowing nitrogen gas. Choose CsBr (99.9%) and PbBr_2_ (99.9%) powders as the deposition materials; after water removal treatment, they were placed in two thermal evaporation crucibles. The vacuum degree of the evaporation chamber is reduced to 3 × 10^−3^ pa, and the thickness of the films and the evaporation rate of CsBr and PbBr_2_ can be monitored by a quartz crystal monitor. To further improve the crystalline quality of the films, the original films were annealed in ambient air (RTP-500) in the range of 400–460 ℃. The PeLEDs have a device structure of Ag (100 nm)/N-type Si/NiO (40 nm)/CsPbBr_3_ (100 nm)/ITO (100 nm). Firstly, CsPbBr_3_ films were deposited on N-type Si (100) substrates by thermal co-evaporation (SD400M). Secondly, NiO films as the hole transport layer were deposited on the CsPbBr_3_ films by magnetron sputtering technique (XSYZKFQ-1200). The sputtering power was 60 W, the gas flow rates of Ar and O_2_ were 45 and 10 sccm, volume ratio was 3 mTorr chamber pressure during sputtering, and the sputtering rate was 0.1 Å/s. ITO and Ag electrodes also use magnetron sputtering technique. ITO electrode was deposited on the surface of the films, and the sputtering power was 100 W, the Ar airflow rate was 40 sccm, and the sputtering rate was 0.6 Å/s. Ag electrode was deposited on the backside of the N-type Si substrate, the sputtering power was 150 W, the Ar gas flow was 40 sccm, and the sputtering rate was 9 Å/s.

Absorption spectra of the CsPbBr_3_ films were recorded using an UV–VIS–NIR scanning spectrophotometer (UV-3101PC). The photoluminescence (PL) spectra were measured by 325 nm He–Cd laser and a fluorescence spectrophotometer (FLS920) as the light source and detector [27]. Before every measurement, the equipment is calibrated to ensure high accuracy and reliability. The crystallinity and phases of films were examined by the X-ray diffraction (XRD, Bruker D8 Advance) pattern. The surface morphologies of thin films were observed by scanning electron microscope (SEM, Hitachi, SU8020).

## Results and Discussion

### Control of Evaporation Rate Ratio

The evaporation rate is a key factor that affects the stoichiometric ratio of the source material and the performance of the device. The competition between how fast atoms on the surface migrate and how quickly the next atoms arrive on the surface and form an immobile cluster with the existing atoms (i.e., deposition rate) is the primary determining factor of the films morphology [28]. Since the co-evaporation method cannot monitor the molar ratio incident on the substrate surface, in order to obtain CsPbBr_3_ films with the correct element ratio, the evaporation rate ratio of the two precursors need to be better adjusted and controlled [13].

**Fig. 1:**
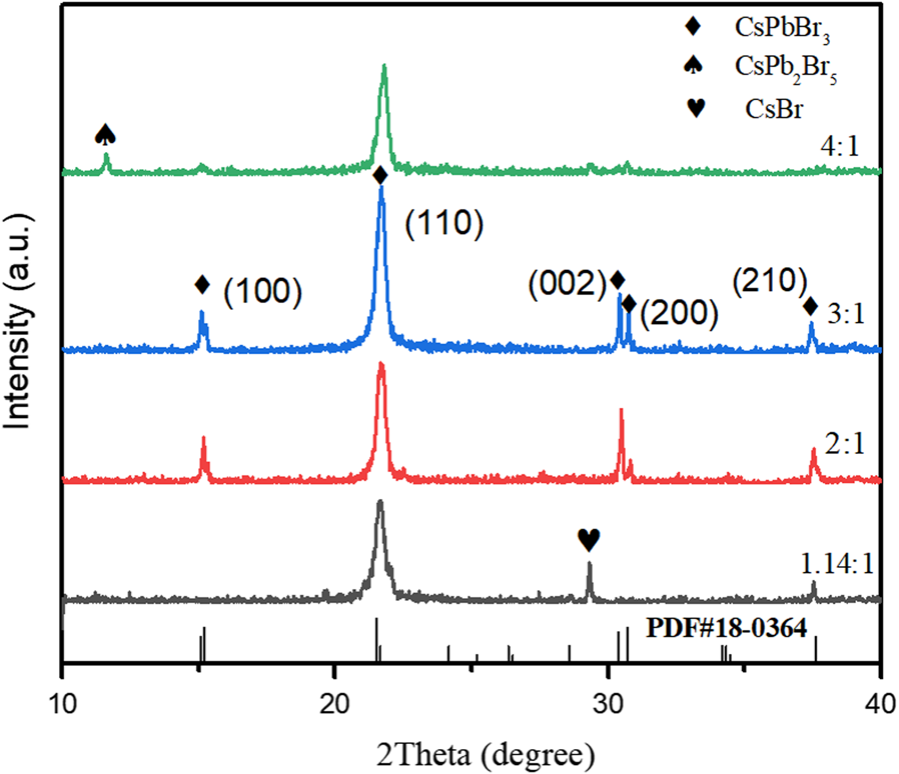
XRD patterns of CsPbBr_3_ films with different evaporation rate ratios

First, we studied the effect of evaporation ratio on the crystal structure of perovskite films. Figure 1 shows the XRD patterns of CsPbBr_3_ films with different evaporation rate ratios (1.14:1–4:1). When the evaporation rate ratio of PbBr_2_ to CsBr is 1.14:1, an additional peak at 29.8°appears, which assigns CsBr phase (PDF#05–0588). It is also found in energy-dispersive spectrometer (EDS) data. As shown in Table 1, when the PbBr_2_ and CsBr evaporation ratio reaches 1.14:1, the atomic percentages of Cs, Pb, and Br in the CsPbBr_3_ films are 31%, 11%, and 58%, respectively. The Pb element percentage is significantly lower than the theoretical value (20%, 20%, 60%), causing by the PbBr_2_ powders loss during deposition process [23].

**Table 1:** Analysis of the element ratio of CsPbBr_3_ thin films prepared under different evaporation rate ratios

Element	Evaporation ratio (PbBr_2_:CsBr)			
	Atomic percentage			
	1.14:1	2:1	3:1	4:1
Br	58.40	62.07	62.43	64.39
Cs	30.80	20.66	18.46	16.22
Pb	10.78	17.27	19.12	19.39
Total	100.00	100.00	100.00	100.00

In order to increase the Pb ratio in CsPbBr_3_ films, we adjusted the evaporation rate ratio of PbBr_2_ to CsBr. When the evaporation rate ratio is 3:1, the peaks at 2θ = 15.1°, 15.3°, 21.5°, 30.4°, 30.7°, and 34.2° should be ascribed to the diffraction of (001), (110), (110), (002), (200), and (210) crystal planes (PDF#18–0364), indicating the formation of the cubic CsPbBr_3_ phase, (110); diffraction peak intensity is stronger for the other ratios, showing that the deposited CsPbBr_3_ films have the best crystallinity. Moreover, the atomic percentages of Cs, Pb, and Br in the CsPbBr_3_ films are 18%, 19%, and 62%, respectively. The corresponding ratio of Cs, Pb, and Br is 1:1.1:3.4, which is the nearest to 1:1:3 of CsPbBr_3_ among all the evaporation rate ratios. However, when the evaporation rate ratios continue to increase, CsPb_2_Br_5_ phase appears, and the element proportion mismatches, as shown in Table 1. As a result, the suitable evaporation rate ratio for CsPbBr_3_ films is 3:1 of PbBr_2_ to CsBr.

**Fig. 2:**
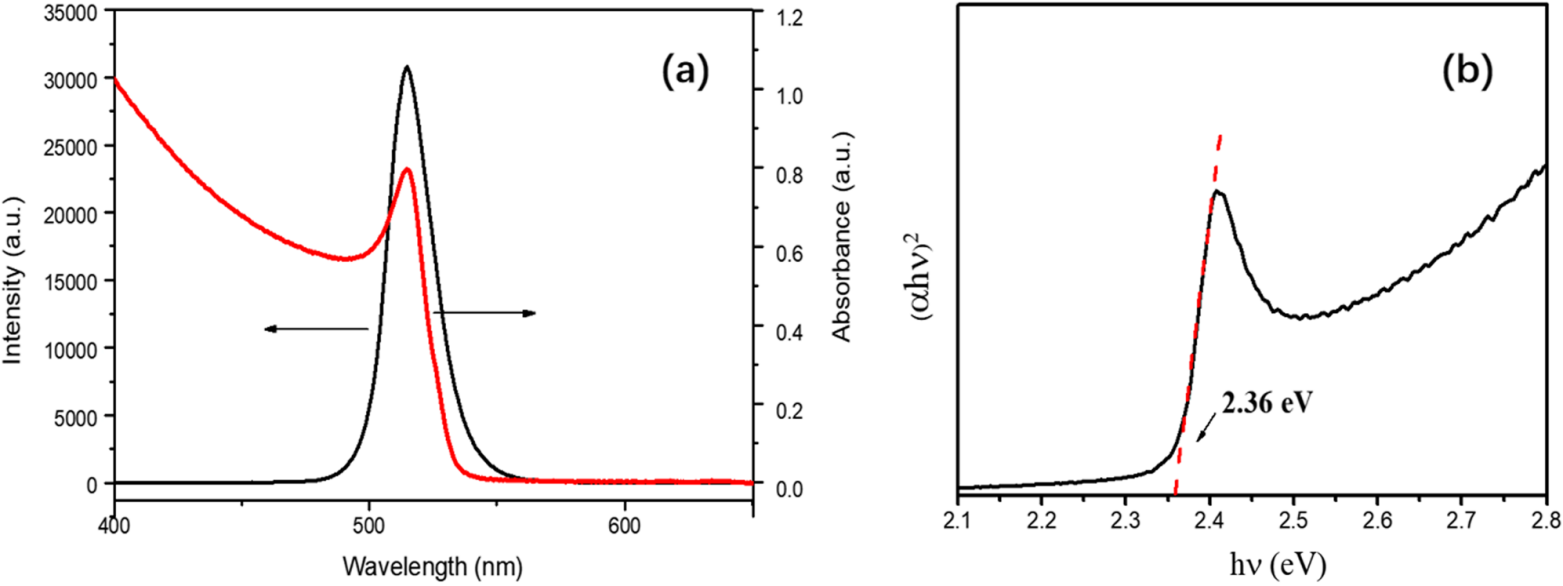
**a** The PL and absorption spectra of CsPbBr_3_ thin films **b** Plot of (αhν)^2^ versus hν of CsPbBr_3_ thin films

Figure 2a shows the optical properties of CsPbBr_3_ films with co-evaporation rate ratio of 3:1, containing the PL and absorption spectrum. The bright green PL is obtained. The luminescence peak position of the films is 517 nm, with full width at half maximum (FWHM) of 18 nm, and an intrinsic light absorption peak appears at the luminescence peak position.

The optical band gap E_g_ of the films can be calculated by transmission spectrum. According to the optical coefficient equation [29, 30]:

$$\left( {h\nu \alpha } \right)^{1/n} = A\left( {h\nu - E_{g} } \right)$$

where $h\nu$ is the photon energy, α is the absorption coefficient, *E*_g_ is the optical band gap, *A* is the proportional constant, and the value of exponent n depends on characteristic of materials, n = 1/2 and 2 for direct and indirect band gaps, respectively. CsPbBr_3_ materials are used as a direct-gap semiconductor, and n should be equal to 1/2. Taking the linear region of hν-(αhν)^2^ curve and drawing the reverse tangent [31], as shown in Fig. 2b, the optical band gap of the films is calculated to be 2.36 eV, which is basically consistent with the other relevant report [32, 33].

### Study on the Substrate Temperature of the CsPbBr_3_ Thin Films

**Fig. 3:**
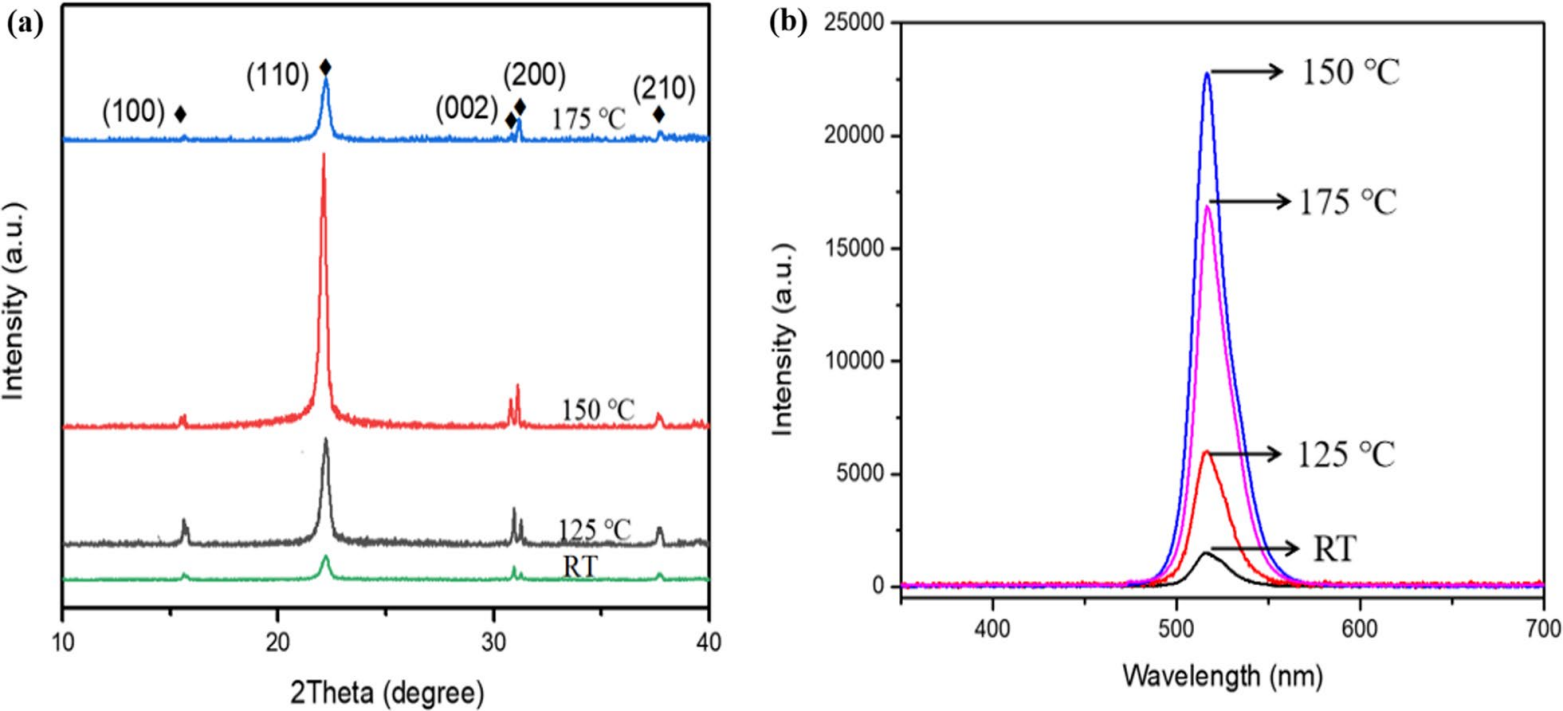
**a** XRD patterns, **b** PL spectra of CsPbBr_3_ films at different substrate temperatures

As for vacuum co-evaporation, the substrate temperature not only affects crystal growth dynamics, but also influences the sticking coefficient, which further changes the migration rate of atoms on the films surface [34]. Moreover, the crystal grain size and morphology of the films are controlled by adjusting the substrate temperature, so as to obtain a smoother surface morphology. We studied the effect of substrate temperature (room temperature (RT) – 175 °C) on the crystallinity of the CsPbBr_3_ films. XRD patterns of CsPbBr_3_ films at different substrate temperatures are shown in Fig. 3a; when the substrate temperature is 150℃, (110) crystal plane exhibits the strongest diffraction intensity and extremely narrow diffraction half-width, indicating that the CsPbBr_3_ films deposited at this temperature have the best crystallinity. Figure 3b depicts optical properties of thin films at different substrate temperatures, including PL spectra. Under 325 nm excitation, the thin films all show an obvious luminescence peak near 517 nm, and the light intensity is the strongest when the substrate temperature is 150℃, demonstrating that this CsPbBr_3_ film shows the highest photogenerated carrier efficiency, which is also consistent with the results obtained from XRD patterns. Therefore, substrate temperature of 150 ℃ is the optimal growth condition of thin films.

**Fig. 4:**
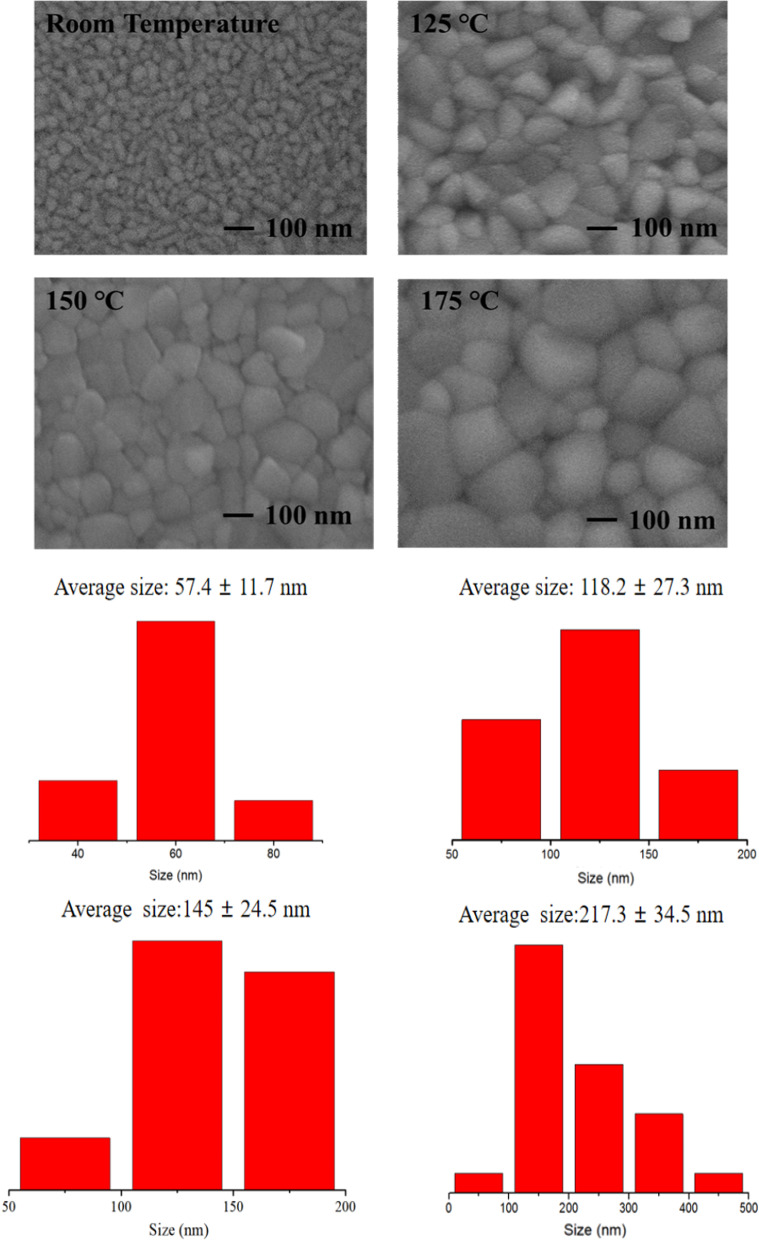
SEM spectra and particle size distribution of CsPbBr_3_ films at different substrate temperatures

Surface morphology of the CsPbBr_3_ thin films at different substrate temperature is exhibited in Fig. 4. It can be seen that increase in the substrate temperature can indeed improve crystallinity and grain sizes of the films. The average grain sizes become much larger, and the films become more compact. These are mainly because the atoms and molecules get higher energy, so they are more likely to migrate and rearrange on the substrate surface and tend to move to the thermodynamic equilibrium position, so the films become more compact. When the substrate temperature reaches 175℃, although the adsorbed atoms have high kinetic energy, the evaporation of atoms on the substrate surface is intensified due to the high temperature, and part of CsPbBr_3_ grains is decomposed, so the uneven grain size leads to the decrease in the crystallinity of the films.

### Effect of the Rapid Annealing on the CsPbBr_3_ Thin Films

#### Annealing Temperature

In order to further improve the grain size and crystallinity of CsPbBr_3_ films, the as-deposited CsPbBr_3_ films (deposited at optimal evaporation rate ratio of 3:1 and substrate temperature of 150 ℃) were submitted to a post-annealing process. To investigate how annealing temperature affects the crystallization of CsPbBr_3_ perovskite, we adjusted the temperature value from 400 to 480 ℃.

**Fig. 5:**
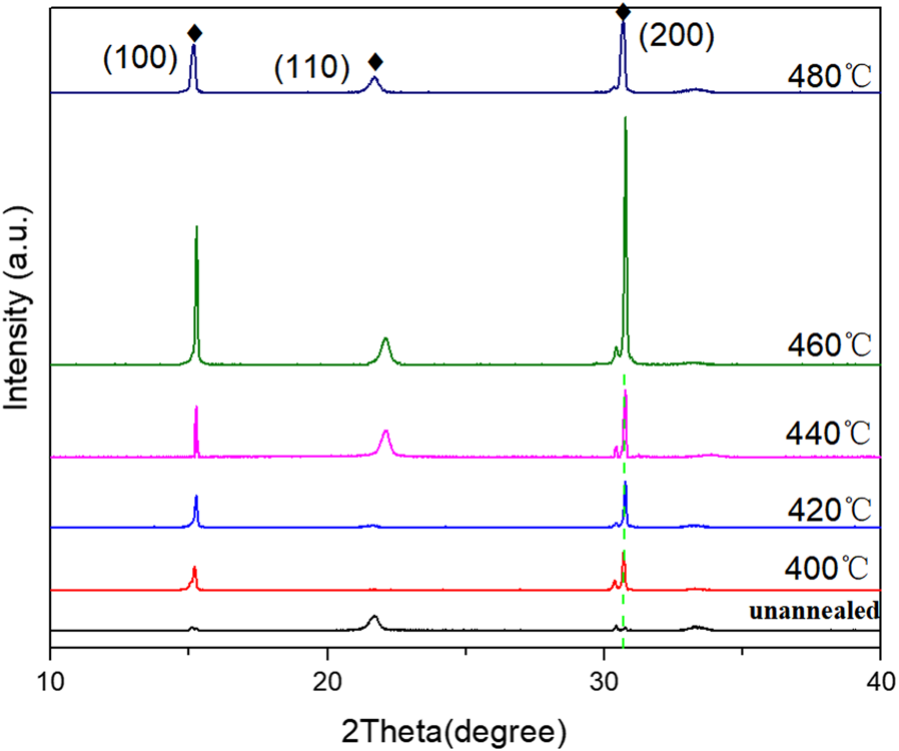
XRD patterns of CsPbBr_3_ thin films annealed at different temperatures

As shown in Fig. 5. The characteristic peaks of CsPbBr_3_ at 2θ = 15.2°, 21.6°, 30.37°, and 30.7° correspond to (100), (110), (002), and (200) crystal planes (PDF#18–0364), respectively, indicating the formation of cubic crystal phase. When the annealing temperature is 460℃, the intensity of diffraction peak is the strongest, indicating that the CsPbBr_3_ films deposited at this temperature have the best crystallinity. During annealing process, the preferential crystal orientation changed from (110) to (100) and (200). Annealing alters the crystallization kinetics of perovskite, driving CsPbBr_3_ films recrystallize, transferring to a more stable structure. In addition, with the increase in annealing temperature, the intensity of XRD diffraction peaks is increased, indicating improved crystallinity of the CsPbBr_3_ films.

During the deposition process, various defects and vacancies in the films will increase. Internal stress generally exists in the thin films, which affects the device performance. At present, it is generally accepted that the internal stress can be divided into thermal stress and intrinsic stress. To put it in detail, rapid annealing mainly influences thermal stress of films. When extra heat is applied to the perovskite, dislocations can compound in the films, crystallinity improved, and the thermal stress in the films reduces. Under high-temperature annealing, the cooling rate is too fast, and the thermal stress is difficult to release. Therefore, annealing to reduce or eliminate the thermal stress in the thin films can improve the crystallinity of the CsPbBr_3_ films and further achieve the higher-performance devices [35–38].

**Fig. 6:**
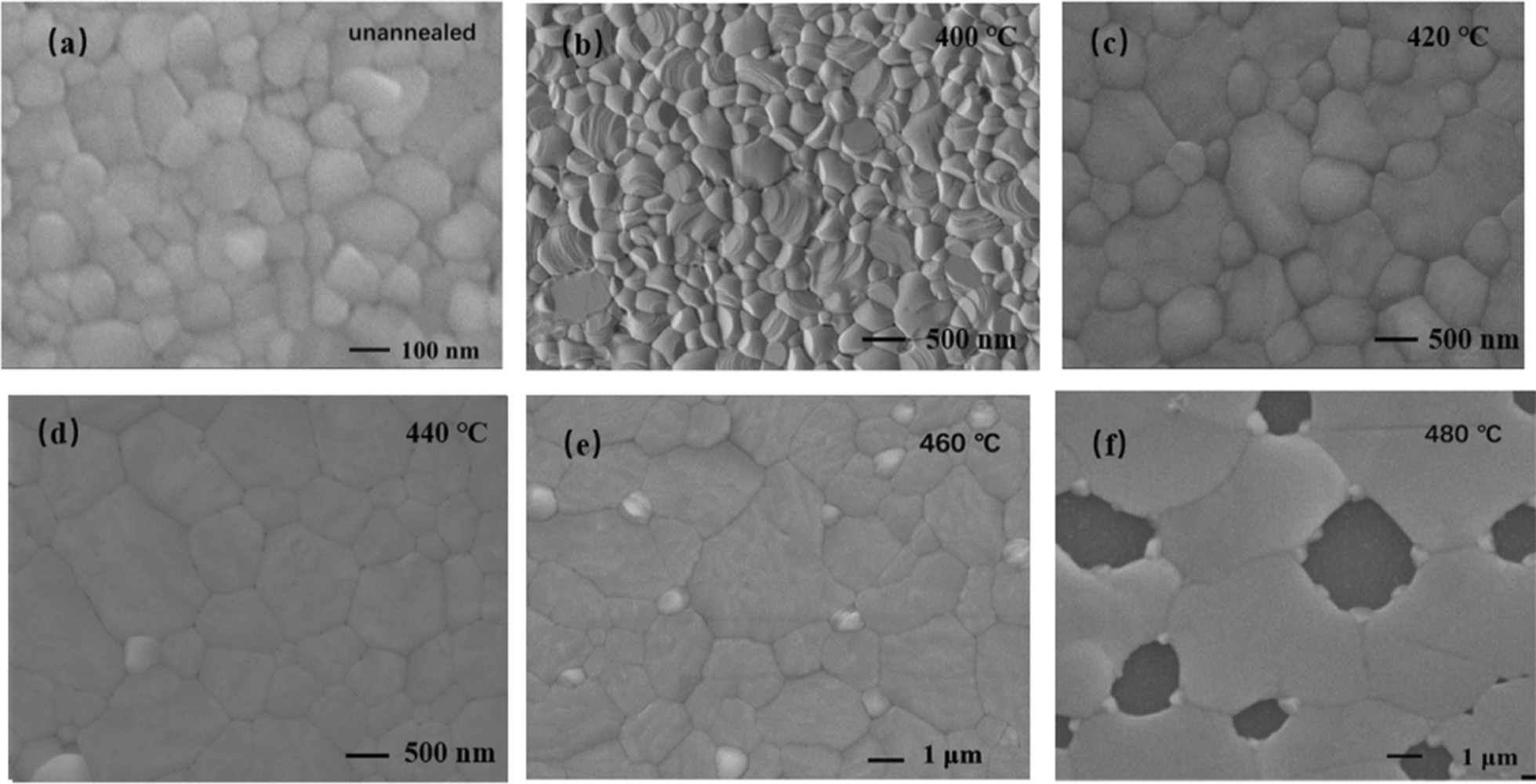
SEM images of CsPbBr_3_ films annealed at different temperatures **a** unannealed, **b** 400℃, **c** 420℃, **d** 440℃, **e** 460℃, **f** 480℃

The impact of the annealing temperature on the films morphology was examined with scanning electron microscope (SEM), as shown in Fig. 6. The CsPbBr_3_ films are composed of dense crystal grains with a complete surface coverage. The as-deposited CsPbBr_3_ films have small grain sizes, with an average grain sizes of 100 nm. When the annealing temperature is 420 ℃, most of the CsPbBr_3_ grains grow up to 400–600 nm, and the films surface becomes flatter. By further increasing the annealing temperature, we find the grain sizes of CsPbBr_3_ continue to grow. When the annealing temperature is 440℃, a large number of CsPbBr_3_ crystals with grain sizes can reach 1 µm, and the pinholes nearly disappear. The increase in grain sizes and the elimination of pinholes are mainly attributed to the external heat energy. The vacancies, interstitial atoms, and dislocations that were originally “frozen” migrate to the surface or grain boundaries and disappear. A large number of non-equilibrium defects disappear so that the thermal stress is released. In addition, when the annealing temperature is high enough, the grains in the films undergone a various recrystallization, which increases the grains and further reduces the grain boundaries [37, 38]. When the annealing temperature is 460 ℃, the CsPbBr_3_ grains are very large (1.5 µm-3 µm). Annealing is beneficial for CsPbBr_3_ films reconstruction and recrystallization. As large grains annex small grains, average grain sizes increase. As a result, the films become flatter and crystallinity improved [39].

**Fig. 7:**
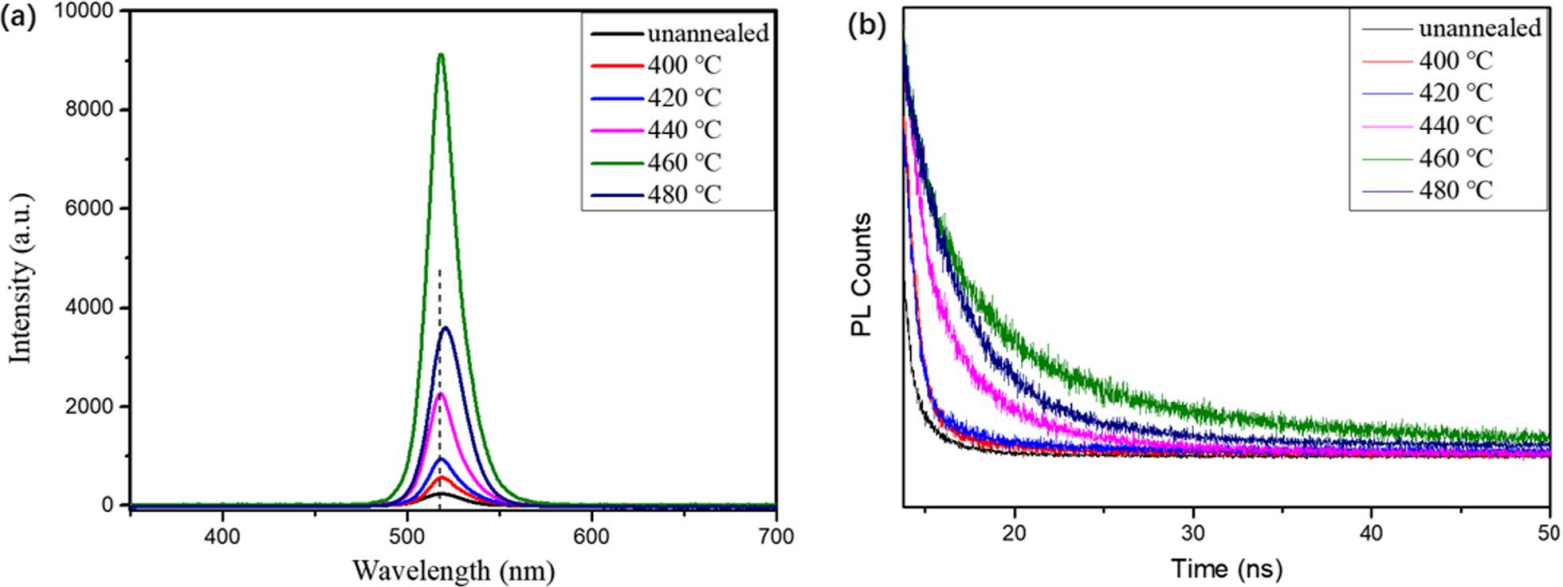
**a** PL spectrum, **b** time-resolved PL decay curves of CsPbBr_3_ thin films annealed at different temperatures

The optical and photoelectric properties of the CsPbBr_3_ films are studied by photoluminescence (PL) spectra, as shown in Fig. 7a. The PL peak position is determined by the band gap width of CsPbBr_3_ films. Therefore, the PL peak of the films appears near 520 nm in the visible region, maintaining the characteristics of direct band gap transition. As the annealing temperature increases, the PL intensity strengthens constantly, but once annealing temperature exceeds 460 ℃, the intensity weakens. The enhanced PL intensity can be attributed to the improved crystallinity of the films. By using bi-exponential decay function, the curves are fitted to determine the fast decay times (τ_1_) and slow (τ_2_) components [40, 41]. Using a bi-exponential decay function *F*(*t*) = *A*_1_exp(− *t*/τ_1_) + *A*_2_exp(− *t*/τ_2_) + *A*_0_ [42]. The average carrier lifetimes of unannealed, 400, 420, 440, 460, and 480 ℃ annealed CsPbBr_3_ films are calculated to be 0.79, 0.90, 0.93, 2.52, 6.3, and 4.0 ns, respectively. The CsPbBr_3_ films annealed at 460 ℃ have the longest carrier lifetime, reflecting the best photoelectric quality, and this also coincides with the analysis of SEM and XRD patterns.

#### Annealing Time

**Fig. 8:**
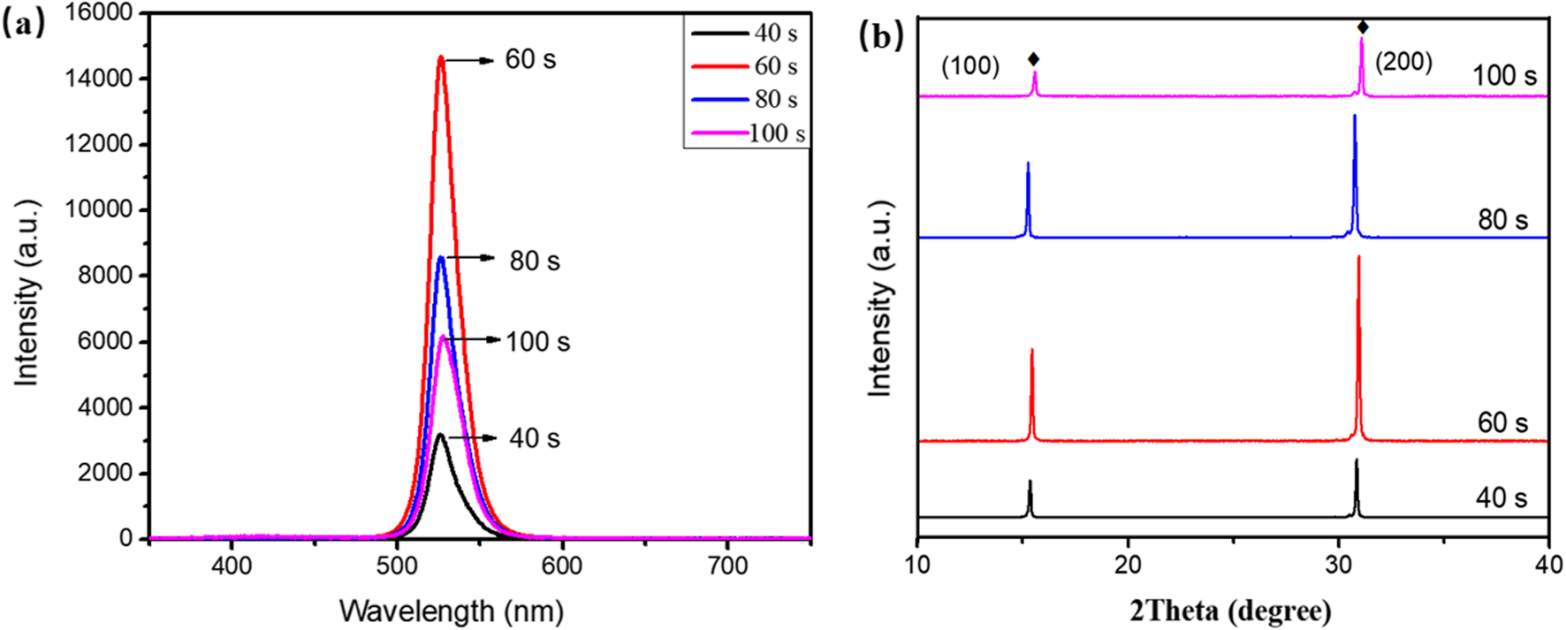
**a** The PL spectra (λex = 325 nm), **b** XRD patterns of CsPbBr_3_ films annealed at 460 ℃ for different times

As previously reported, the effect of the annealing conditions on CsPbBr_3_ films was investigated, including annealing temperature. Figure 8a shows the PL spectra of CsPbBr_3_ thin films annealed at 460 ℃ for various times from 40 to 100 s in ambient air. Appropriate annealing time is beneficial to improve the grain sizes and photoelectric quality of thin films. As the annealing time increases from 40 to 60 s, the luminescence intensity increases continuously and then decreases. XRD patterns as shown in Fig. 8b and the diffraction intensity of (100) and (200) phases both enhance, and once the annealing time above 60 s and the intensity both recede, the results are consistent with PL intensity.

**Fig. 9:**
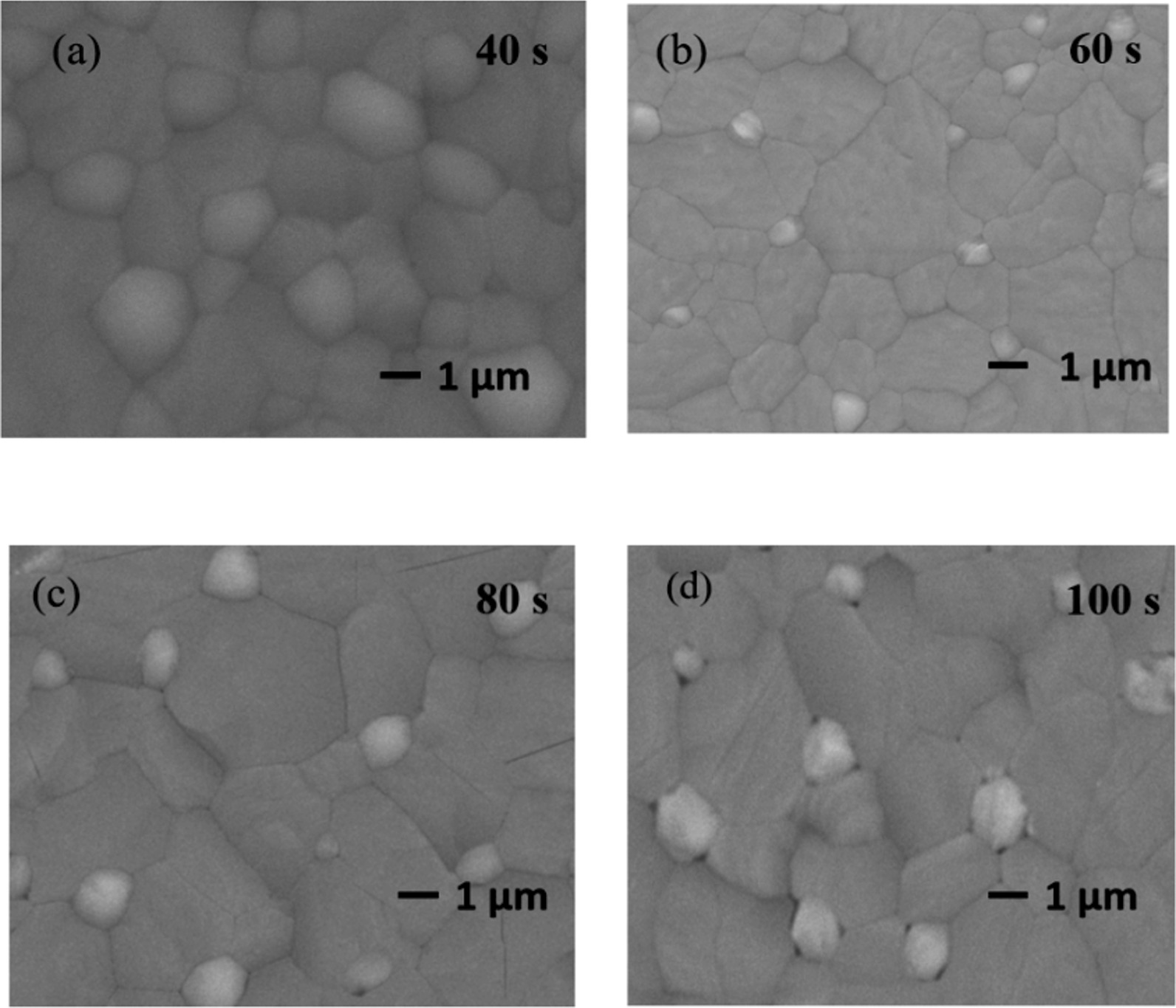
SEM images of CsPbBr_3_ films annealed at 460 ℃ for different times

To further explore the morphology of the films, Fig. 9 shows the SEM images under different annealing times, and the average grain sizes range from ~ 2 to ~ 3.4 μm. When the annealing time is 60 s, the average grain sizes can reach 2.5 μm. Proper annealing time is helpful to improve the crystallinity of the films. But too long annealing time leads to the grains decomposition, and the element ratio does not meet the requirement of 1:1:3, which reduces PL intensity. So the best annealing condition is at 460 ℃ for 60 s, which can achieve the strongest PL intensity and the best crystallinity with large grains. These are also consistent with the results obtained by PL spectra and XRD patterns.

## Research on the Stability of CsPbBr_3_ Thin Films

**Fig. 10:**
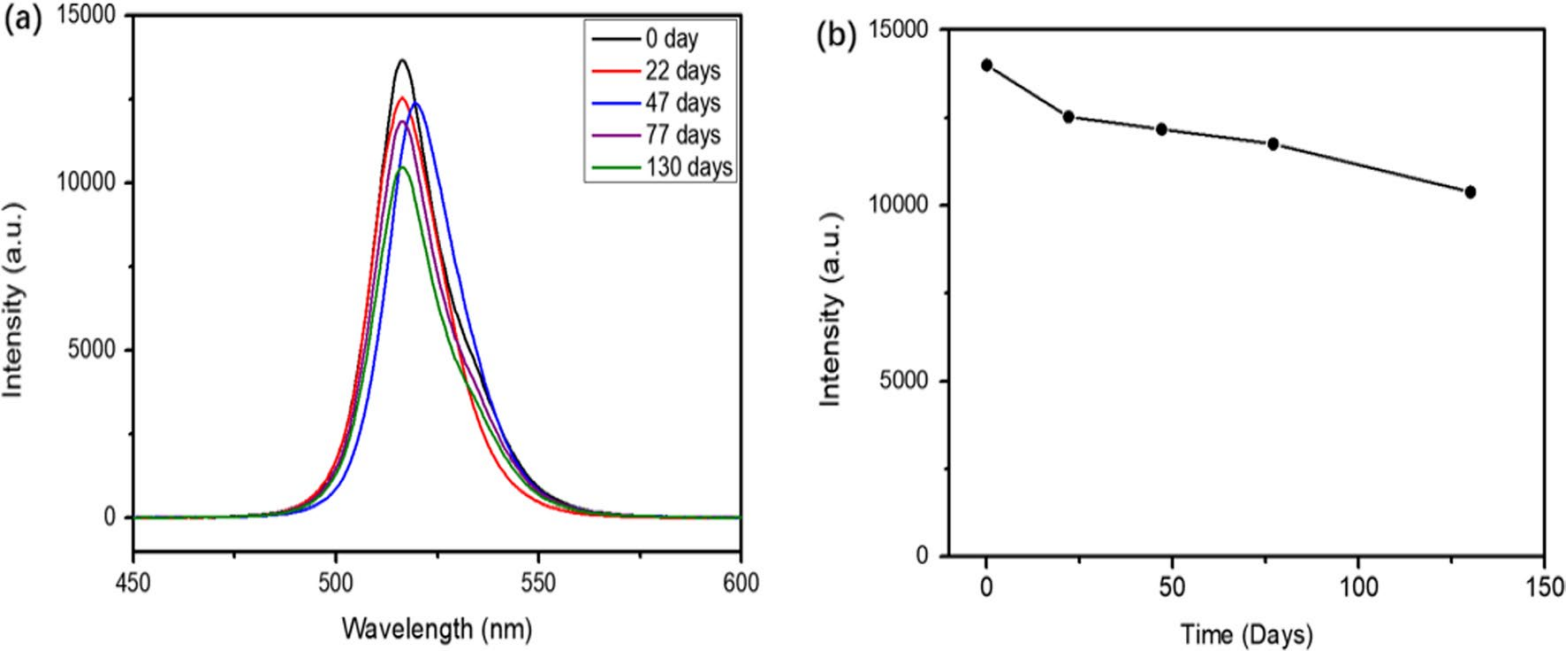
**a** Peak position of the PL spectrum with different storage times in ambient air (λex = 325 nm). **b** The PL intensity trend

In order to verify the stability of all inorganic halogen perovskite and further optimize the films quality, the annealed samples were placed at room temperature in air atmosphere, the PL intensity was measured about 130 days, as shown in Fig. 10, it can be seen that the PL intensity did not decay so much, and the decay rate was about 13% in the first 75 days. Tiny variation of peak position was assigned to CCD’s instrumental error. The effect of long-term storage on peak position was not obvious, and the peak position was still at 517 nm. The PL intensity and peak position of the films remained stable. Therefore, the preparation of CsPbBr_3_ films by thermal evaporation method and annealing has good stability, which have great potential in improving device performance.

### Study on the PeLEDs

**Fig. 11:**
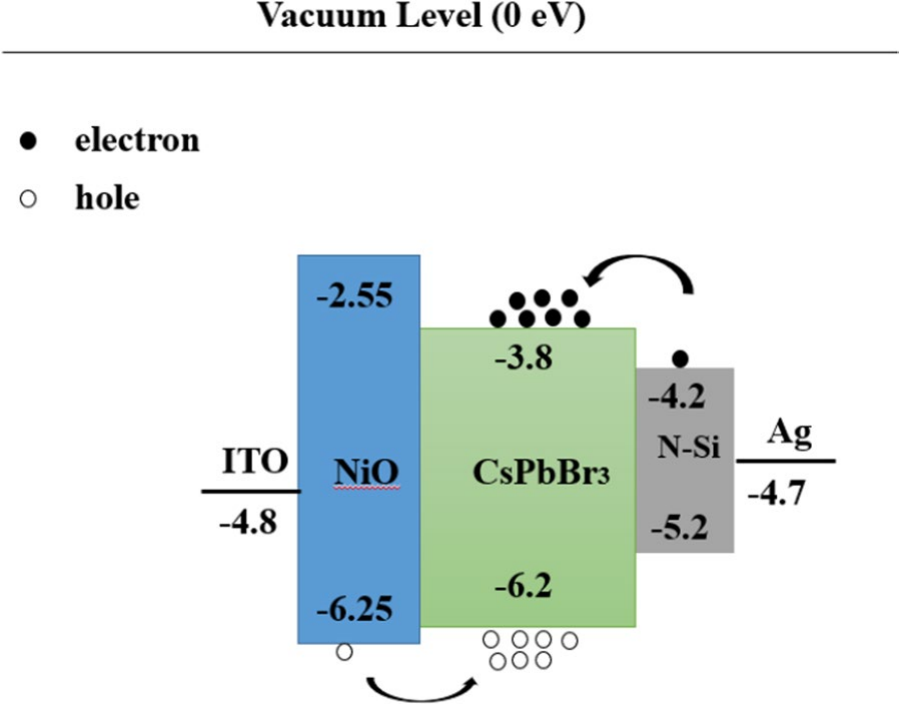
The working mechanism of the PeLEDs based on the energy band diagram

**Fig. 12:**
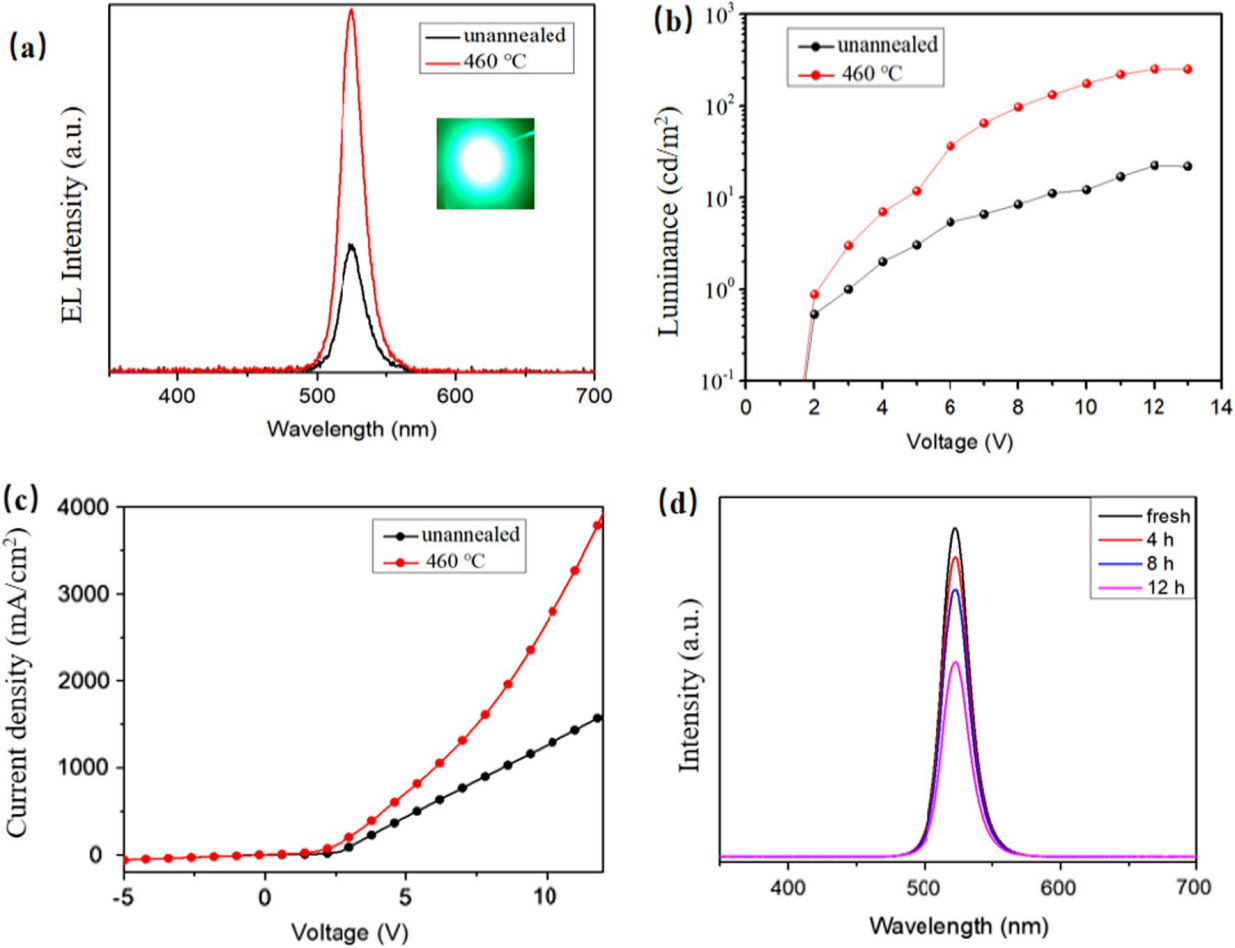
**a** EL spectra, **b** luminance against voltage (*L*–*V*) curves, **c** current density against voltage (*J*–*V*) curves of PeLEDs, and **d** EL spectra acquired at different operating cycles under the same bias and measurement conditions

Based on the best crystallinity and morphology, PeLEDs were fabricated. The devices architecture is shown in Fig. 11, and perovskite CsPbBr_3_ layer is the emitting layer of carrier radiation recombination. The working mechanism of the device can be discussed according to the most basic physical progresses of light-emitting diodes [4, 43, 44]. Driven by forward voltage, green electroluminescence of the device is observed under dark conditions, the PeLEDs exhibit good rectification characteristics, the reverse current is small, and the turn-on voltage of the device is ~ 3 V. Figure 12a presents the EL spectrum of the devices, and inset figure is the photograph of our PeLEDs at an injection voltage of 12 V. The spectra show an emission peak at 520 nm with the FWHM of 18 nm, which indicates our PeLEDs have an excellent color purity. Additionally, the devices with annealed CsPbBr_3_ films show higher EL intensity compared to the unannealed films, which indicates that the surface morphology and crystallinity of the films are helpful to improve the performance of the devices. After annealing, the devices performed best with a maximum luminance (*L*_max_) of 252 cd m^−2^. The results in both *L*_max_ and EL intensity thus emphasize the importance of films morphology and coverage on device performance. Moreover, the PeLEDs can work continuously for 12 h, and the emission attenuation is ~ 30% in ambient air, with relatively superior stability.

## Conclusion

In summary, we have successfully prepared CsPbBr_3_ thin films by thermal co-evaporation. We studied the effects of co-evaporation rate ratio, substrate temperature, and annealing conditions on the properties of the films. We found that the optimal deposition conditions of CsPbBr_3_ films were substrate temperature of 150 ℃ and evaporation rate ratio of PbBr_2_ to CsBr of 3:1, and as-deposited films had high crystallinity and excellent stability. Furthermore, overall films showed the obvious luminescence peak near 517 nm, with narrow FWHM. In addition, by introducing the annealing process, we found that with increasing annealing temperature and time, the films tended to be flatter with larger grain sizes. The PL intensity of CsPbBr_3_ films also had variation tendency, which were consistent with XRD and SEM analysis. The best annealing condition was at 460 ℃ for 60 s, which could achieve the strongest PL intensity and the best crystallinity with large grains up to 3 μm. Once exceeding this value, PL intensity and crystallinity of CsPbBr_3_ films began to decline. The obtained films with large grain sizes and high quality have a very broad application prospect. These provide a basis for further large-scale production of stable, efficient, and all inorganic CsPbBr_3_ optoelectronic devices. The corresponding LEDs had a very good color purity, which could work continuously for 12 h in ambient air, with relatively superior stability.

## Data Availability

All data generated or analyzed during this study are included in this published article.
